# Evaluation of How Methacrylate Gelatin Hydrogel Loaded with *Ximenia americana* L. Extract (Steam Bark) Effects Bone Repair Activity Using Rats as Models

**DOI:** 10.3390/jfb14090438

**Published:** 2023-08-23

**Authors:** Seânia Santos Leal, Gustavo Oliveira de Meira Gusmão, Valdiléia Teixeira Uchôa, José Figueiredo-Silva, Lucielma Salmito Soares Pinto, Carla R. Tim, Lívia Assis, Antonio Luiz Martins Maia-Filho, Rauirys Alencar de Oliveira, Anderson Oliveira Lobo, Adriana Pavinatto

**Affiliations:** 1 Scientific and Technological Institute, Brazil University, São Paulo 08230-030, Brazil; seaniasantos@gmail.com (S.S.L.); carla.tim@universidadebrasil.edu.br (C.R.T.); livia.assis@universidadebrasil.edu.br (L.A.); 2 Biotechnology and Biodiversity Research Center, State University of Piauí, Teresina 64002-150, Brazil; figueredo_silva@hotmail.com (J.F.-S.); lucielmasalmito@ccs.uespi.br (L.S.S.P.); almmaiaf@ccs.uespi.br (A.L.M.M.-F.); 3 GrEEnTeC-PPGQ, State University of Piauí—UESPI, 2231 João Cabral Street, P.O. Box 381, Teresina 64002-150, Brazil; gomgusmao@ccn.uespi.br; 4 PPGQ-GERATEC-CD, State University of Piauí, Teresina 64002-150, Brazil; valdileiateeira@ccn.uespi.br; 5 School of Health Sciences, State University of Piauí, Teresina 64002-150, Brazil; rauirysalencar@ccs.uespi.br; 6 Interdisciplinary Laboratory for Advanced Materials (LIMAV), Materials Science & Engineering Graduate Program (PPGCM), Federal University of Piauí (UFPI), Teresina 64049-550, Brazil

**Keywords:** biomaterials, plant extract, hydrogel, bone repair, photobiomodulation

## Abstract

The use of bioactive materials, such as *Ximenia americana* L., to stimulate the bone repair process has already been studied; however, the synergistic effects of its association with light emitting diode (LED) have not been reported. The present work aims to evaluate the effect of its stem bark extract incorporated into methacrylate gelatin hydrogel (GelMA) on the bone repair process using pure hydrogel and hydrogel associated with LED therapy. For this purpose, the GelMA hydrogel loaded with *Ximenia americana* L. extract (steam bark) was produced, characterized and applied in animal experiments. The tests were performed using 50 male Wistar rats (divided into 5 groups) submitted to an induced tibia diaphyseal fracture. The therapy effects were verified for a period of 15 and 30 days of treatment using histological analysis and Raman spectroscopy. After 15 days of induced lesion/treatment, the new bone formation was significantly higher in the GXG (GelMA + *X. americana* L.) group compared to the control group (*p* < 0.0001). After 30 days, a statistically significant difference was observed when comparing the GXLEDG (GelMA + *X. americana* L. + LED) and the control group (*p* < 0.0001), the GXG and the control group (*p* < 0.001), and when comparing the GG, GXG (*p* < 0.005) and GXLEDG (*p* < 0.001) groups. The results shows that the *Ximenia americana* L. stem extract incorporated into GelMA hydrogel associated with LED therapy is a potentiator for animal bone repair.

## 1. Introduction

Bone repair is a natural process that involves a series of complex biological events aimed at restoring bone integrity and function. Some factors that can affect bone repair include age, nutrition, blood supply, stability, diabetes, smoking and certain medications [1]. In cases of severe fractures or complications, bone grafts or growth factors can be used to stimulate bone repair. Although bone grafts can be effective in promoting bone regeneration, there are several challenges and limitations associated with this approach, such as limited availability, healing time, graft resorption, non-union and late union, risk of infection, immune response, rejection and nonviable cost [2,3]. Given the challenges associated with conventional approaches, researchers and healthcare professionals are continually exploring alternative treatments for bone repair. Some promising options include tissue engineering, including structures, growth factors and stem cells [3,4,5,6]; bone morphogenetic proteins (BMPs) [7]; mesenchymal stem cell therapy [6]; 3D printing [4,5,8]; and gene therapy [7,9].

Photo-crosslinkable hydrogels with exceptional biocompatibility and biodegradability possess the capacity to substantially enhance cell migration, proliferation and differentiation, making them extensively employed in the field of tissue engineering [8,10]. Methacryloyl gelatin (GelMA) is a flexible hydrogel derived from gelatin and modified with methacrylamide and methacrylate groups, allowing it to crosslink when exposed to light irradiation. This versatile hydrogel can be fabricated using diverse methodologies, including microforming, photomasking, bioprinting and microfluidic techniques. It is characterized by high flexibility, porosity and hydrophilicity, leading to minimal inflammatory responses when used in the body. In addition, they exhibit promising osteoinductive properties that are beneficial for bone repair [11,12,13]. Their structure closely resembles the cell matrix, making them well suited for cell culture both in vitro and in vivo [14].

In the same context, natural compounds derived from medicinal plant extracts, such as *Davallina orientalis*, *Lepidium sativum* L., Cimicifuga racemose (Actaea), *Piper sarmentosum*, *Ormocarpum cochinchinense*, *Peperomia pellucida*, *Symphytum officinale*, Chenopodium ambroisioides L., *Epimedium sagittatum*, *Nigella sativa*, *Aloe vera*, *Sambucus williamsii*, *Ulmus davidiana*, *Spinacia oleracea*, *Dalbergia sissoo*, *Marantodes pumilum*, *Cassia occidentalis* L. [15], Astragalus membranaceus [16], Anredera cordifolia [17], Cissus quadrangularis, Withania somnifera and Tinospora cordifolia [18], are widely used to promote bone repair. Among the most studied herbal medicines, *Ximenia americana* L., which is a plant that is found in various regions of the world, possesses medicinal properties that are utilized and recognized [19]. The pulverized stem of *Ximenia americana* L. has been recommended for the repair and control of the inflammatory process, and proven results in research carried out through experimental models showed that this extract can be used to promote tissue repair [20]. The aqueous crude extract derived from various parts of the plant, including the leaves, roots, stem and stem bark, contains a range of secondary metabolites. These include tannins, flavonoids, saponins, steroids and triterpenes [21,22,23].

Phytochemical investigations of *Ximenia americana* L. steam bark reported by Santana et al. [19] and Almeida et al. [24] have revealed the presence of several bioactive compounds in different extracts, including aqueous, ethanolic and hydroalcoholic extracts. These compounds include condensed tannins, hydrolysable tannins, saponins, glycosides, polyphenols, flavonoids and terpenoids [23]. Flavonoids play a crucial role in various physiological processes within the body. They aid in the absorption of iron and vitamins and exhibit several beneficial properties, including antioxidant, antimicrobial, immunomodulatory, anti-inflammatory, and antinociceptive effects [25]. In addition, flavonoids possess analgesic properties and contribute to the regeneration of cartilage [26] and bones [27,28,29,30]. They also promote vasodilation and tissue healing in incisional wounds. In the context of bone repair, flavonoids exhibit anti-osteoclastic and anti-inflammatory effects by inhibiting the expression of osteoclastic markers, reducing reactive oxygen species and pro-inflammatory cytokine levels, as well as matrix metalloproteinases. Additionally, flavonoids enhance the osteogenic potential of pre-osteoblastic cells and stimulate the overexpression of osteogenic markers [29,31]. The flavonoids found in *Ximenia americana* L. are catechin, rutin, myricetin and (-)-epicatechin, the latter being found in greater amounts in the stem and root extracts [32,33,34].

The application of photobiomodulation LED therapy (light emitting diode therapy) has been widely studied in the treatment of different bone conditions [35,36,37,38]. The results of these studies have shown that the LED therapy in the near-infrared (invisible) wavelength spectrum has a positive effect on bone tissue metabolism and on fracture consolidation [39,40,41,42], as it stimulates mitochondrial metabolism, resulting in increased differentiation and proliferation of osteogenic cells and subsequent higher bone matrix deposition [43,44]. This evidence emphasizes the ability of LED therapy to stimulate bone formation and optimize bone repair.

Recent research demonstrates the association of poly (β-aminoester) (PBAE) hydrogel with total flavonoids with osteogenic properties [45]. Given this perspective, the interest in the subject is justified by the scarcity of studies that investigate the incorporation of *Ximenia americana* L. extract into GelMA hydrogel, as well as the association of the components (GelMA and GelMA/extract of *Ximenia americana* hydrogel) with LED (photobiomodulation) in bone repair. Therefore, in the present study, the synergistic effect from pure GelMA hydrogel loaded with aqueous extract from *Ximenia americana* L. stem-bark, and the effect of the hydrogel combined with LED therapy was evaluated.

## 2. Materials and Methods

### 2.1. Ximenia americana L. Extract

A sample of *Ximenia americana* L. was collected in Domingos Mourão (4°09′14.8″ S and 41°18′28.3″ W), a city in Piauí, Brazil, in January 2018. The different parts of the plant, including the stem, leaves, flowers and fruits, were identified and documented. A voucher specimen was preserved under the accession number HAF 03541 at the Herbarium Afrânio Gomes Fernandes (UESPI).

Following the methodology proposed by Carvalho et al. in 2020 [33], the bark of the *Ximenia americana* L. stem (300 g) was washed with water and placed in a beaker containing 2 L of distilled water. The beaker was then stored at 4 °C for 5 days. After this period, the liquid was filtered, and the aqueous extract was obtained. A portion of the extract was lyophilized, and both the aqueous and lyophilized samples were kept frozen until further use.

### 2.2. Incorporation of Ximenia americana L. in GelMA Hydrogel

The photopolymerizable methacrylate gelatin (GelMA) was obtained following the procedure described by Nichol et al. [46]. Briefly, 10 g of Type A gelatin (from pork skin, Sigma-Aldrich, Sao Paulo, Brazil), were dissolved in 100 mL of phosphate buffer solution (pH 7.4, Sigma-Aldrich), mixed and stirred for 1 h at 50 °C. Next, 3 mL of methacrylate anhydride 3-(trimethoxysilyl) propyl methacrylate (Sigma-Aldrich) was slowly dropped and stirred into the system for 3 h at 50 °C. Separately, 400 mL of PBS was pre-warmed to 50 °C and then mixed into the initial solution (reaching a volume of 500 mL). Next, the solution was then dialyzed using deionized (DI) water (12–14 KDa dialysis membranes, Sigma-Aldrich) for 7 days at 40 °C. The deionized water was changed twice a day. Finally, the solution was transferred to Falcon tubes, frozen at −80 °C and lyophilized, and the GelMA was obtained.

The GelMA/*Ximenia americana* L. hydrogel was obtained by adding *Ximenia americana* L. aqueous extract at a concentration of 5% in GelMA solution before the photoinitiator agent Irgacure insertion at a concentration of 0.5% 2-hydroxy-4′-(2-hydroxyethoxy)-2-methylpropiophenone (from Irgacure 2959, Sigma-Aldrich) to a 10% gelatin solution. Eppendorf tubes (used as a template) were taken to UV photocrosslinking (360–480 nm) for 5 min.

### 2.3. Hydrogel Characterization

The spectra were obtained from KBr pellets (spectroscopic grade), at a ratio of 1:100 sample/KBr. Before analyses, all samples (*Ximenia americana* L., GelMA and GelMA/*Ximenia americana* L.) and KBr were dried at 50 °C for 40 min. Next, the tablets were prepared using a press. The analyses were carried out using a Thermo Nicolet Nexus 470 equipment with Fourier transform infrared spectroscopy (FTIR) while using a transmittance module with an accumulation of 48 scans from 4000 to 500 cm^−1^, observing a resolution of 2 cm^−1^.

Thermogravimetric analysis curves (TGA) were obtained on a SDT Q600 analyzer model (TA Instruments, São Paulo, Brazil). TGA curves were obtained using alumina sample support, and approximately 8 ± 0.1 mg of sample was used. The analyses were performed with a heating rate of 10 °C min^−1^ in an atmosphere composed of air and N2, using a flow rate of 40 mL min^−1^.

Differential scanning calorimetry (DSC) curves were obtained in a DSC Q20 calorimeter model (TA Instruments), using an aluminum pan containing 2 ± 10 mg of sample in a nitrogen atmosphere under a flow rate of 20 mL min^−1^. All results were obtained using the temperature ranging from 20 to 800 °C at a heating rate of 10 °C min^−1^. All data analyses were performed using the TA Instruments Universal Analysis 2000 software, version 4.7A.

X-ray diffraction measurements were performed on the GelMA hydrogel samples, including those containing *Ximenia americana* L. and those without it. The samples were ground, the spectra acquisition time increased, and the scan was performed from 10° to 70°, with a step of 0.0200/s and speed of 0.5°/min for a total of 2 h of analyses for each sample. X-ray diffraction analyses were performed in a Rigaku X-ray unit, last model IV 2Theta/Theta, 40 kV voltage and 30 mA current, and a sealed Cu tube was used.

### 2.4. Controlled Release Test of Epicatechin—Main Compound of Ximenia americana L.

UV-Vis (Shimadzu UV-160A, Barueri, Brazil) absorption spectra were performed from 200 to 700 nm to identify the absorbance band of epicatechin, the main compound of the *Ximenia americana* L. sample. An analytical curve, observing the λ max. = 278 nm, was obtained from the *Ximenia americana* L. stock solution (1 mg/mL) in concentrations ranging from 50 to 250 µg/mL. All curves were performed in triplicate (n = 3).

The analytical curve constructed was then used to determine the concentration of epicatechin release through an in vitro release assay of the GelMA epicatechin. For this purpose, the experiment was carried out using the GelMA + *Ximenia americana* L. 5% hydrogel (m = 0.0005 g) was inserted in 10 mL of PBS (pH 7.4 ± 0.1) and incubated at 37 °C under 100 rpm of stirring. In the periods fixed at 15 min, 30 min, 1 h, 2 h, 24 h and for 30 days (maximum experimental time in the in vivo tests for the hydrogel), 3.0 mL of solution was removed from these mediums, and the amount of the epicatechin was detected using UV-Vis spectroscopy. Each experiment was performed in triplicate.

### 2.5. In Vivo Study

#### 2.5.1. Ethical and Legal Aspects, and Experimental Animals

The present study was approved by the Animal Ethics Committee of the State University of Piaui (protocol 00089.007021/2021-66). Fifty male Wistar rats (Rattus norvegicus albinus), 8 weeks old and weighting 250–300 g, were kept at the Animal Hospital located at the State University of Piaui. The animals were housed in standard polyethylene cages under controlled conditions, including a temperature of 24 ± 1 °C, humidity of 60% and a 12/12 h light/dark cycle. They were provided with unrestricted access to suitable food and water.

The animals were divided into five groups (n = 10), with 5 rats from each group euthanized at the experimental times of 15 and 30 days. The groups were submitted to the following:

- Control Group (CG): induced fracture and no treatment;
- GelMA Group (GG): induced fracture and GelMA as treatment;
- GelMA + LED Group (GLEDG): induced fracture and GelMA + LED as treatment;
- GelMA/*Ximenia americana* L. Group (GXG): induced fractures and GelMA/*Ximenia americana* L. as treatment;
- GelMA/*Ximenia americana* L. + LED Group (GXLEDG): induced fractures and GelMA/*Ximenia americana* L. + LED as treatment.

#### 2.5.2. Surgical Procedure

A pre-treatment using atropine provided by Alergan^®^ (Guarulhos, Brazil) was administrated to the animals (0.04 mL/100 g of animal weight). After 15 min, they were anesthetized intramuscularly using 10% ketamine hydrochloride and 2% xylazine hydrochloride provided by Syntec^®^ (0.1 mL/100 g of animal weight) [47,48]. Next, the animals were submitted to depilation and asepsis in the right tibia region with topical polvidone (Bioquímica^®^, Belo Horizonte, Brazil).

The procedures for fracture and implantation of the biomaterial were performed using the protocol adapted by Kido 2015 et al. [49]. Briefly, the critical bone defect was induced after a longitudinal incision in the skin, and the separation of the subcutaneous connective tissue was performed with a surgical micromotor (3 mm in diameter), followed by insertion of the biomaterial at the injury site, according to the treated groups. To obtain this cavity, a constant torque of 45 N was stipulated at a speed of 45,000 rpm and abundant irrigation with saline solution for the viability of bone regeneration.

At the end of the procedure, the animals received subcutaneous tramadol hydrochloride analgesic (12.5 mg/Kg), administered every 6 h. Periodic assessments (every 2 h), were also performed to identify pain by facial expression and behavior. The euthanasia of the animals was proceeded in the period of 15 and 30 days after induction of injury by overdose administrating 150 mg/kg of sodium thionembutal [50,51]. Samples of bone tissue from the groups were removed and kept in liquid nitrogen. The collected samples were sent for Raman spectroscopy analysis and histopathological analysis.

#### 2.5.3. LED Therapy

To stimulate bone repair, 1 LED light device (Endophoton, KLD, Biosistemas, Amparo, Brazil) was used, which emitted in the near infrared electromagnetic spectrum of 858 ± 20 nm. The intervention of the LED group was performed after the surgical procedure, which corresponds to the period from the beginning of the inflammatory phase of bone repair for several days after the postoperative period until euthanasia. The application was performed on the right tibia using the punctual technique (one point on the fracture), with the equipment pen positioned perpendicularly to the bone tissue for 120 s using 6 J of energy, 12 J/cm^2^ of energy density and 0.1 W/cm^2^ of potency.

#### 2.5.4. Histological Analysis

Buffered formalin was used for 48 h to fix each sample, and after fixation, the samples were decalcified with EDTA (ethylendiaminetetra acetic acid, 10% *w*/*v*, pH 7.2) for four weeks. After decalcification, the samples were immersed in alcoholic solutions (gradually increasing concentrations) for dehydration and treated with xylene in an automatized tissue processor (PT05 TS Luptec, Sao Carlos, Brazil). After being embedded in paraffin, a rotating microtome (MRP09 Luptec, Sao Carlos, Brazil) was used to obtain serial histopathological sections (with a thickness of 5 μm and a distance between 2 and 3 μm,), and the samples were colored using hematoxylin and eosin (H.E.) at two sections/blade.

The samples were studied using a trinocular light microscope (model Olympus CX31, Tokyo, Japan), and photographic images were made in triplicate using a digital camera (Moticam WiFi X, MoticMicroscopes, Richmond, VA, USA) with connection to a computer. The histopathological semiquantitative analysis of new bone formation was performed according to the aspects described in the literature [52,53]. All images were enlarged using a micrometric ruler as a parameter of magnitude of amplification, which was inserted into all images collected using 4× and 10× magnification objectives. The processed specimens were evaluated through comparative descriptive analysis.

#### 2.5.5. Dispersive Raman Spectroscopy

To perform the Raman spectroscopy, the samples were kept at room temperature (removed from the nitrogen). The spectra were obtained in a Raman spectrometer (model Senterra II, Bruker, Fällanden, Switzerland), using λ = 785 nm laser for excitation. The laser parameters used are 50 mW of output power, a spectral resolution of 9–15 cm^−1^ for 15 s, a gamma spectral set up at 400 a, and a 90-3, 500 cm^−1^ 10× objective. The spectrum was collected in triplicate (10 μm of distance between the points) for all bone regions of interest. The spectrum of normal cortical bone, referred to as healthy, was acquired from a region distant from the induced lesion after euthanizing the animals at 15 and 30 days. To compare the Raman spectra of the biomaterials (GelMA and GelMA + *Ximenia americana* L.), with the treated and healthy regions, the raw spectrum in the range of 90–3500 cm^−1^ was processed using the Labspec 5.0 program. Fluorescence was eliminated by applying a fifth-order polynomial fit, and additional preprocessing steps, such as baseline adjustment, were performed.

The obtained spectra were normalized using a normalization vector. This normalization process involved dividing the Raman intensity by the square root of the sum of the calculated square intensities of the entire spectrum. The normalization was performed using Origin 2018 software. After identifying the peaks in the spectra, the integrated areas of the main evaluated peaks were calculated within the range of 957–962 cm^−1^, which corresponds to the phosphate band and is representative of the mineral content in the bone. This analysis aimed to quantify the bone composition and assess any alterations in the mineral.

To further characterize bone alterations in the mineral, crystallinity was determined by calculating the inverse of the full peak width at half maximum (FWHM) of the υ1 phosphate band peak, which is located around ~960 cm^−1^. The FWHM represents the width of the peak at its half maximum intensity, and by inverting this value, the crystallinity of the bone can be obtained. This provides insights into the degree of mineralization or structural changes in the bone sample.

#### 2.5.6. Statical Analysis

The analysis of the results was conducted using GraphPad Prism^®^ software (version 8.3.0, Instat Software Inc., La Jolla, CA, USA). Statistical data analyses were performed using ANOVA as a parametric test and the Kruskal–Wallis test as a non-parametric test. A Dunn post-test was applied for further analysis. The significance level was set at *p* < 0.005, indicating statistical significance. The results are presented as means and standard deviations, providing a measure of central tendency and variability, respectively.

## 3. Results and Discussion

### 3.1. Production and Characterization of Raw Materials and GelMA and GelMA + Ximenia americana L. Hydrogels

**Figure 1:**
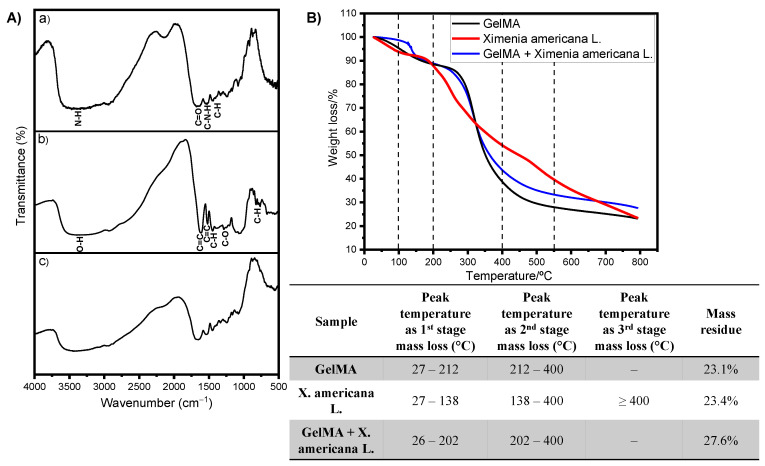
(**A**) FTIR spectra in vibrational regions from 4000 to 500 cm^−1^ for *Ximenia americana* L. (**a**), GelMA, and (**b**) GelMA + *Ximenia americana* L. (**c**). (**B**) Thermal analysis results.

FTIR spectroscopy was performed to characterize the material and evaluate its chemical composition. Figure 1A presents the spectra for the GelMA, *Ximenia americana* L. and GelMA + *Ximenia americana* L. Figure 1A(a) presents the *Ximenia americana* L. spectrum. The following are main bands found and attributed at approximately 3000 to 3800 cm^−1^ band, related to the O-H stretching bond at 1614 and 1517 cm^−1^ bands assigned to the C=C bonds of the aromatic groups; at 1448 and 1388 cm^−1^ bands, related to C-H bonds; at 1300 to 1000 cm^−1^ bands, characteristic of C-O stretching vibration; and at 796 and 667 cm^−1^ bands, corresponding to C-H bonds in aromatics.

The bands were assigned according to the results found [19,24]. Figure 1A(b) presents the GelMA spectrum. The main bands found and assigned were around 3000 and 3600 cm^−1^ bands, referring to the peptide bonds (N-H); at 1650 cm^−1^ bands, corresponding to Amide I, mainly the C=O stretching groups; and at 1490 and 1580 cm^−1^ bands, corresponding to C-N-H folding vibrations. The studies by Santana et al. [19] and Almeida L et al. [24] provided insights into the chemical bonds associated with various functional groups, including ethers, esters and carboxylic acids. These functional groups are commonly found in flavonoids, tannins, anthraquinones and other secondary metabolites present in the extract. The information from these studies helped in identifying and interpreting the specific chemical bonds observed in the spectra. Figure 1A(c) showed that the GelMA + *Ximenia americana* L. spectrum maintained the same profile of the GelMA spectrum, only observing an increase in the transmittance intensity of the bands. This fact demonstrates that there was an overlapping of the bands of the GelMA and GelMA + *Ximenia americana* L. materials and that this overlap is not indicative of chemical incompatibility.

The X-ray diffractograms of the *Ximenia americana* L. samples and the GelMA + *Ximenia americana* L. hydrogel show a high degree of amorphization, and only a very wide band can be seen for both samples in the region between the angles of 10° and 40°, corresponding to pure gelatin [54].

Thermogravimetric results for GelMA, *X. americana* L. and GelMA + *X. americana* L. are shown in Figure 1B. As can be observed, the results for *X. americana* L. showed three decomposition stages, with a mineral residue of ~23.4%. The first event occurs in the temperature range between 27 and 138 °C and can be attributed to the loss of volatile materials, such as the water used in the process. On the other hand, the second and third events occurred at temperatures above 200 °C and 400 °C, respectively, and can be associated with the decomposition of a wide variety of secondary metabolites, mainly the phenolics present in the extract. Equal results were obtained by Santana et al. [19], and by Almeida et al. [24]. The thermal degradation behavior of the GelMA shows two mass loss events: The first occurs in the temperature range between 27 and 212 °C, probably caused by the loss of water molecules, and a second event occurs at temperatures higher than 325 °C, which can be attributed to the degradation of the biopolymer, with a residue of ~23.1% of mass. Similar thermal behavior results were obtained by Aldana et al. [55]. The thermal behavior for the GelMA + *X. americana* L. hydrogel is similar to that obtained for the GelMA. As can be observed, there are two mass loss events for the sample: the first is observed in the temperature range between 26 and 202 °C caused by the loss of water molecules and volatile materials, and the second event occurs at temperatures above 200 °C, which can be attributed to the degradation of the biopolymer and the decomposition of secondary metabolites, and a residue of approximately ~27.6% by mass was observed. Based on the results of the TG/DTG analysis, we can infer that both the GelMA and GelMA + *X. americana* L. have good thermal stability at temperatures up to 200 °C with no significant mass loss (decomposition) in this temperature range. This result enables the application of these materials in this temperature range. More information regarding the thermal behavior of the samples were obtained in the DSC analysis. In the DSC curve of the GelMA, an endothermic band was observed in the range between 30 and 120 °C, and that can be attributed to the glass transition of blocks of amino acids in the peptide chain relative to the amorphous regions of gelatin and to the loss of water and protein breakdown. A similar result was found by Aldana et al. [55] and El-Maghawry et al. [56]. The DSC curve for the GelMA + *X. americana* L. hydrogel shows an endothermic band in the range of 36–110 °C that can be attributed to the glass transition of amino acid blocks in the peptide chain relative to the amorphous regions of gelatin and to the loss of water and degradation of proteins, as seen in the GelMA DSC curve. In the region between 250 and 320 °C, an endothermic band was observed, which can be attributed to the breakage of hydroxyl bonds present in *X. americana* L. A similar phenomenon was observed by De Salvi et al. [57].

### 3.2. Controlled Release Test of Epicatechin—Main Compound of Ximenia americana L.

**Figure 2:**
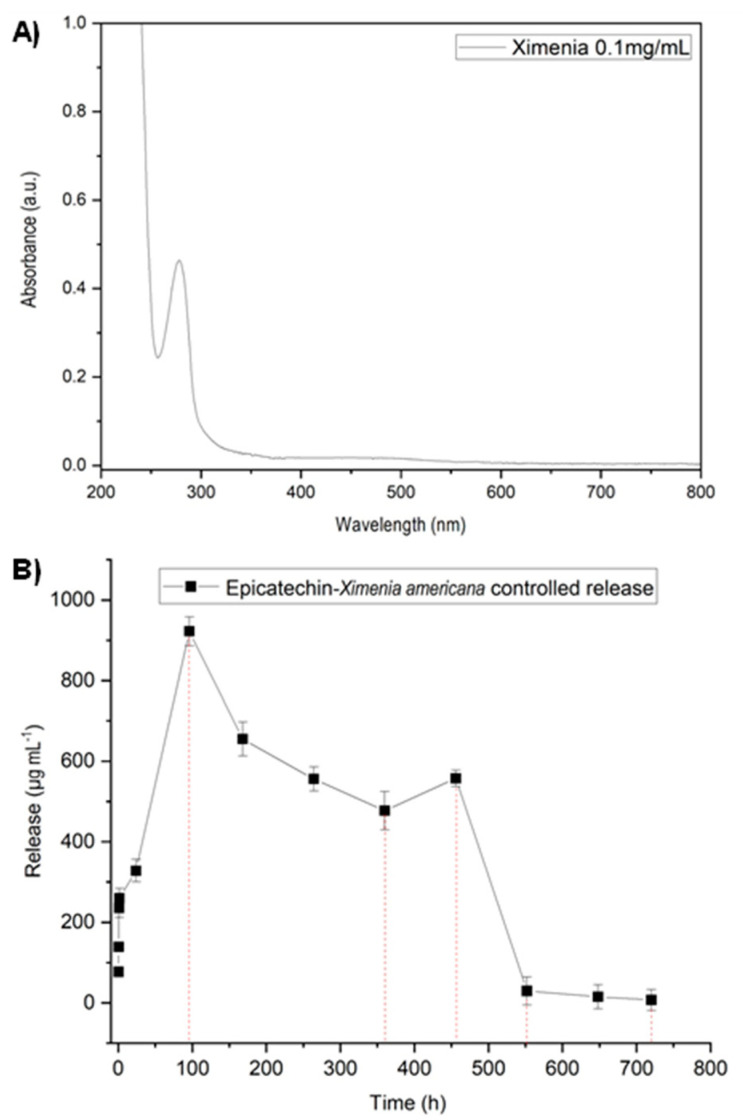
(**A**) UV-Vis spectrum of the *Ximenia americana* L. extract. (**B**) Controlled release of epicatechin as a function of time from the GelMA + *Ximenia americana* L. hydrogel.

The biochemical characterization of *X. americana* L. in most existing studies is concentrated on the investigation of the pulp and seed of the fruit, with rare studies involving the stem, even knowing that this component is widely used in traditional medicine. Figure 2A shows the UV-Vis spectra of the *Ximenia americana* L. stem extract, and a peak centered at 278 nm was observed. According to a study published by Aragão [58], absorption bands centered at 270 nm, 278 nm and 280 nm correspond to the main constituents of the *X. americana* L. stem bark, which are procyanidin B, catechin/epicatechin and procyanidin C, respectively. Among these compounds, epicatechin was found in greater amounts in studies published by Santana et al. [19]. Both studies reported the presence of bands referring to epicatechin between 276 nm and 278 nm. Based on these studies, the band found at 278 nm from the *X. americana* L. extract was attributed to the epicatechin/catechin compound (C_5_H_14_O_6_).

Next, to evaluate the epicatechin release time by the GelMA + *X. americana* L., the analytical calibration curve and the controlled release of epicatechin were performed. The obtained analytical curve showed a linear range from 5 to 250 μg/mL, and the interval can be expressed by the equation y = 0.00000296x + 0.001 (R^2^ = 0.998). Figure 2B presents the release assay results from the GelMA + *X. americana* L. hydrogel. As can be observed, the release of the extract starts at ~15 min after application and continues upwards until reaching the maximum release peak (922.50 µg/mL^−1^) after ~4 days. After that, the concentration released starts to decline slowly until ~15 days, and a new release peak occurs at ~19 days (654.9 µg/mL^−1^), decreasing quickly at ~23 days (0.8296 µg/mL^−1^). The amount released at the experimental time of 30 days is minimal, constant and close to zero (0.233 µg/mL^−1^). By not zeroing out and continuing to release even in smaller amounts until the 30th day of the experimental period, it can be concluded that the release of *X. americana* L. is adequate during the period in which the in vivo tests were carried out.

### 3.3. In Vivo Experiments

**Figure 3:**
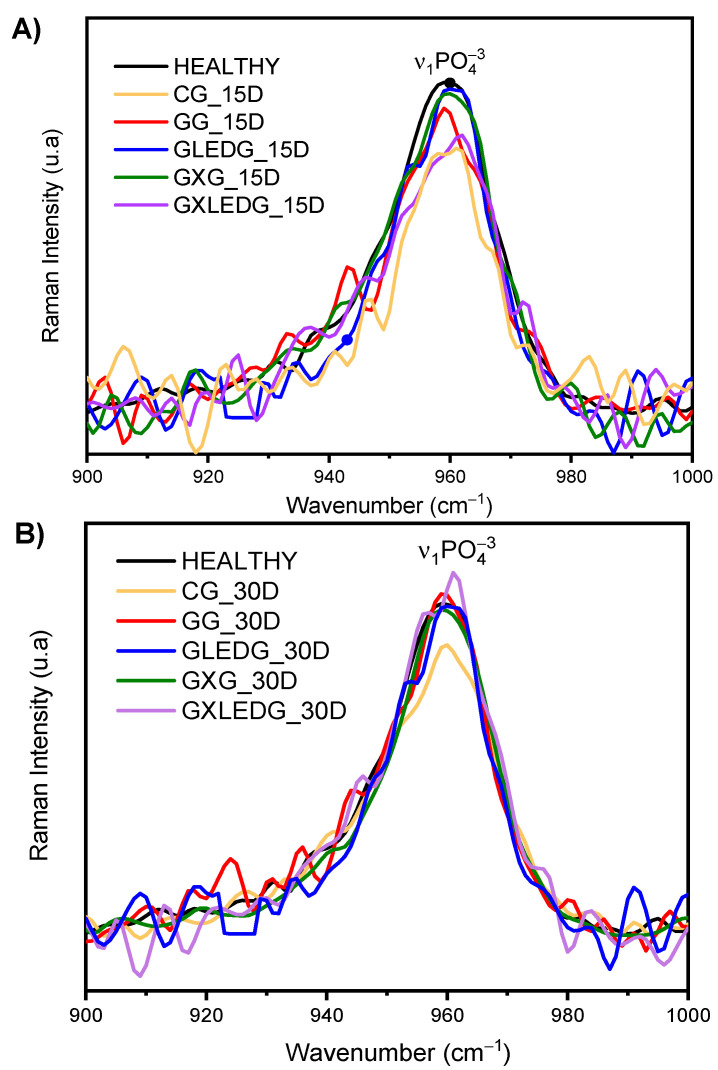
Mean Raman spectra of the bone tissue for the groups at (**A**) 15 days and (**B**) 30 days after the surgical procedure at ~960 cm^−1^, referring to υ1 PO_4_^3−^.

Bone healing can indeed be monitored using Raman spectroscopy since it allows for the determination of hydroxyapatite concentration/incorporation, which is an essential component of mineralized bone. Hydroxyapatite is a calcium phosphate mineral that constitutes a major part of the inorganic bone matrix. Through this technique, the hydroxyapatite phosphate band, centered at approximately 960 cm^−1^, can be monitored [59], and used as a bone repair marker [60,61,62]. Figure 3 A,B shows the Raman spectra for 15 and 30 days after the surgical procedure, respectively. As can be observed in Figure 3A, the GLEDG (GelMA + LED) and GXG (GelMA + *X. americana* L.) groups showed bands with greater intensity and closer to the healthy one, suggesting an optimized bone regeneration process in these groups, with a high deposition of υ1 PO_4_^3−^ in a concentration similar to that of healthy bone. On the other hand, the CG showed a lower level of mineralization than the others, indicated by the low relative intensity of the band associated with υ1 PO_4_^3−^. The greater bone repair observed for the mentioned groups can be explained by the immediate effect of using *X. americana* L. and LED in the GXG and GLEDG groups, respectively. Regarding GXG, we can attribute this optimization of bone repair to the large amount of epicatechin released by the *X. americana* L. in the first days (presented in Figure 2B), where a peak maximum of release was observed at ~4 days. Epicatechin is a flavonoid, which presents antioxidant activity attributed to the phenolic radicals of its structure. These findings corroborate the study by Wan Osman et al. [63], which investigated the use of noni leaves (rich in epicatechin), in bone repair and in combating inflammation of the joint cartilage in rats. The investigation took place through cultures of cartilage explants and preclinical studies. In their results, in a period prior to 30 days, epicatechin suppressed the release of glycosaminoglycan and nitric oxide from the cartilage explant and significantly reduced the amount of mRNA in joint tissues, thus increasing bone formation in addition to improving the structure of joint cartilage and chondrocytes. Regarding the GLEDG (GelMA + LED) group, the improvement can be attributed to the systemic and instantaneous effect resulting from photobiomodulation through LED irradiation to the tissue. Photobiomodulation from LED increases bone metabolism and accelerates fracture healing. Our results corroborate findings in other research studies [38,59,64], which have also found evidence of the optimization of bone repair in fractures in a period prior to 30 days.

From the Raman spectra presented in Figure 3B, it is possible to observe that the relative intensity of the υ1 PO_4_^3−^ band for the GG, GLEDG and GXG groups has the same intensity to that found for healthy bone. Interestingly, the intensity of the band found for the GXLEDG group exceeded what was shown for healthy bone, suggesting a greater deposition of phosphate (bone) than normal. This result corroborates the large amount of newly formed bone around the fracture shown for these groups, as seen below in the histological analysis.

It is known that low intensity laser therapy (LILT) and photobiomodulation have the main purpose of promoting the interaction between biological tissues associated with their optical characteristics, such as reflection, transmission, scattering and absorption [65]. Regarding the effects of photobiomodulation on bone tissue, the therapy aims to stimulate cell proliferation since this resource has the capacity to interact with bone content, favoring the biochemical modulation of bone cells, stimulating mitochondrial respiration, accelerating osteogenic potential through the migration and differentiation of these cells to the irradiation site, stimulating collagen production and the mineralization of the extracellular matrix [66]. On the other hand, the CG presented a lower level of mineralization than the other groups, which was expressed by the low relative intensity of the υ1 PO_4_^3−^ band in the same experimental period (30 days).

**Figure 4:**
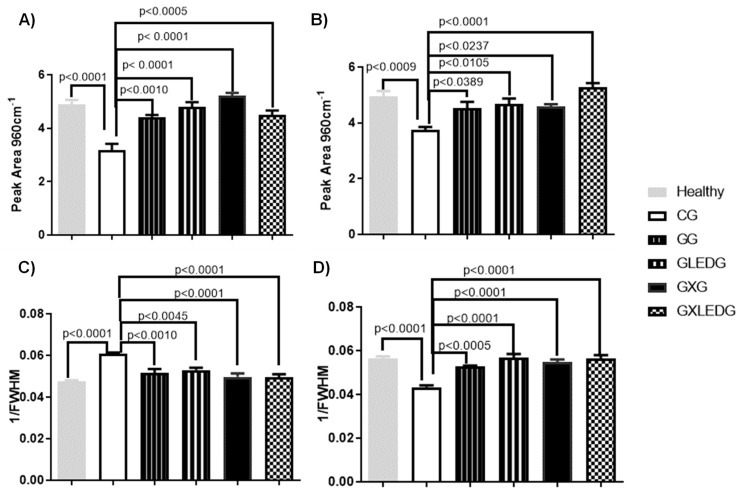
Mean and standard deviation of the integrated areas for bands centered at 960 cm^−1^ (related to υ1 PO_4_^3−^) after 15 (**A**) and 30 days (**B**) of treatment. Mean and standard deviation of crystallinity after 15 (**C**) and 30 days (**D**) of treatment.

Figure 4 A,B presents the spectra of the integrated areas of the band centered at 960 cm^−1^ (related to υ1 PO_4_^3−^) after 15 and 30 days, respectively, which is related to the carbonated apatite data. On the 15th day of the experiment, a statistical difference was observed when comparing all treated and control groups, suggesting an increase in PO_4_^3−^ deposition that is statistically significant (*p* < 0.0001) between the GLEDG (GelMA + LED) and GXG (GelMA + *Ximenia americana* L.) groups, with *p* < 0.001 for the GXLEDG (GelMA + *Ximenia americana* L. + LED) and GG (GelMA), confirming the findings in Figure 3. A difference (*p* < 0.05) was also found between the GG (GelMA) and GXG (GelMA + *Ximenia americana* L.) groups, also noting that the control group had a significantly lower PO_4_^3−^ deposition than healthy bone (*p* < 0.0001). After a period of 30 days, a statistical difference comparing all treated and control groups was observed, identifying an increase in PO_4_^3−^ deposition, being statistically significant (*p* < 0.0001) between the GXLEDG (GelMA + *Ximenia americana* L. + LED) and control groups, and *p* < 0.05 between the GLEDG (GelMA + LED), GXG (GelMA + *Ximenia americana* L.) and GG (GelMA) groups. A difference (*p* < 0.05), was also found between the GG (GelMA) and GXLEDG (GelMA + *Ximenia americana* L. + LED) groups, also noting that the control group had a lower PO_4_^3−^ deposition than healthy bone (*p* < 0.001).

Figure 4 C,D presents the spectra for the crystallinity given for the inverse of the width at half height of the peak after 15 and 30 days, respectively. Such data evaluate bone composition by the deposition of new hydroxyapatite crystals in newly formed bone. The crystallinity found in the treated groups (*p* < 0.0001), and of the healthy bone (*p* < 0.001), was significantly higher than that of the control group after 30 days of treatment, with no statistical difference being observed comparing the treated groups. At 15 days of treatment, a statistical difference was observed comparing the healthy group and CG (*p* < 0.05), where the CG had lower crystallinity. The mineral crystallinity results obtained from techniques such as Raman spectroscopy provide valuable information about the size and maturation of mineral crystals in bone tissue, as mentioned in refs. [22,60]. Reduced crystallinity indicates the presence of younger and smaller mineral crystals at the injury site. During the early stages of bone healing, the rapid mineralization process leads to the formation of smaller crystals with lower levels of organization. These crystals may have a higher proportion of non-stoichiometric substitutions and a less defined crystal lattice structure, resulting in reduced crystallinity values. As the bone healing progresses and the tissue matures, the mineral crystals undergo a process of growth and maturation. This is characterized by the replacement of carbonate ions and the incorporation of additional mineral components, leading to larger and more ordered crystals. The increased crystallinity reflects the improved organization and structure of the mineral crystals, indicating a higher degree of maturation [59].

**Figure 5:**
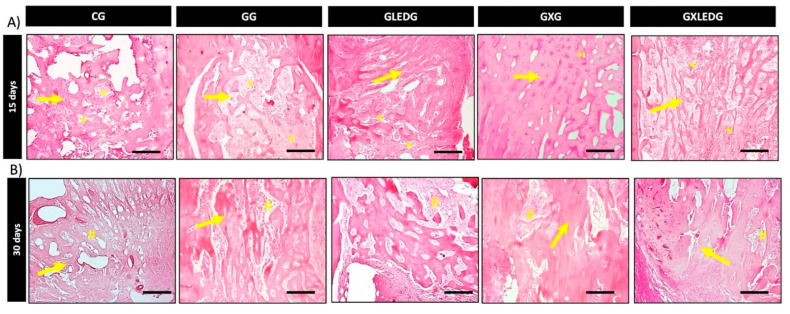
Bone defect photomicrography 15 days (**A**) and 30 days (**B**) after the surgery. Granulation tissue (*); osteoid (#); neoformed bone tissue (→). Bar scale = 500 μm (2.5× image); bar scale = 50 μm (40× image). Used stains: hematoxylin and eosin.

Figure 5 provides a representative histological overview of all the experimental groups at two different time points, specifically 15 days (A) and 30 days (B) after the bone defect was created. After 15 days, for the CG, GG, GLEDG and GXLEDG groups, the presence of granulation tissue and a slight new bone formation can be observed in the entire defect. However, the GXG group presented a greater presence of new bone tissue sites when compared to the other experimental groups.

After 30 days, it was possible to observe that for the CG, GG and GLEDG groups, the center of defect was occupied by osteoid tissue and immature newly formed bone cells, exhibiting some interconnected trabeculae. For the GXG and GXLEDG groups, woven formed bone demonstrated a more mature aspect with a well-arranged bundle of bone tissue compared to the other treated groups.

**Figure 6:**
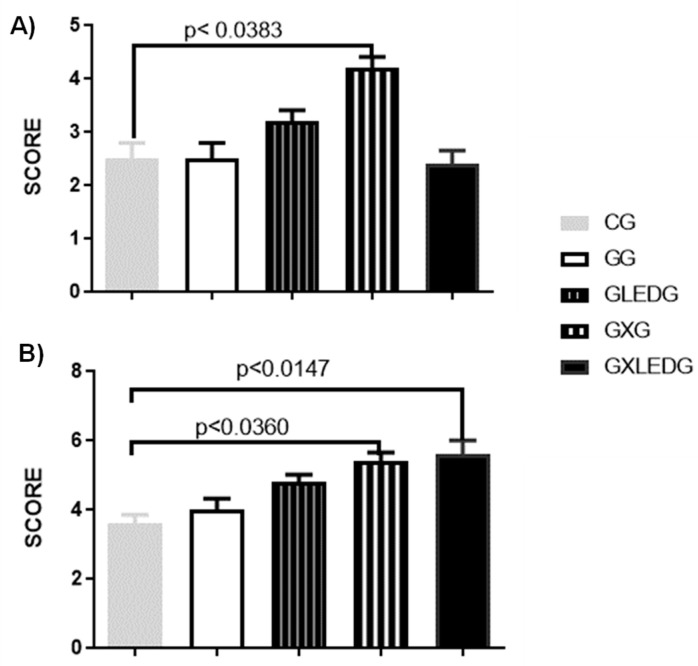
Experimental groups bone repair score 15 (**A**) and 30 days (**B**) after the surgery.

Figure 6 A,B demonstrates the semi-quantitative results of bone repair. In the analysis of the samples at 15 days after surgery, the bone repair score of the GXG group was significantly higher compared to the CG. However, no significant difference comparing the score results for the GG, GLEDG, and GXLEDG groups was observed 15 days after surgery.

Thirty days post-surgery, the GXG and GXLEDG groups had the highest bone repair score when compared to the CG. No other differences were observed in the other experimental groups.

From the findings of the histological analysis, it is possible to suggest that both therapies employed (associated or isolated) provided greater bone trabeculae filling, with the results of bone repair being more expressive in the GXG group at 15 days and in the GXG and GXLEDG groups at 30 days caused by the increased synthesis of osteoblasts and collagen. There are no studies in the literature that have reported the use of *Ximenia americana* L. in bone repair. However, these positive results can be explained by two factors. The first corresponds to the *Ximenia americana* L. itself since several authors [21,22] infer that the stem, as characterized in the present study, has secondary metabolites, such as flavonoids. These metabolites can exert antimicrobial and modulating activities, in addition to anti-inflammatory action, among others [67]. All these properties are inherent to *Ximenia americana* L. when used alone or in association with hydrogels and seem to favor bone repair.

The second factor would be due to the associated treatments, since both the GelMA [68], and photobiomodulation seem to contribute to increased cell metabolism and, consequently, to bone repair. GelMA increases bone tissue due to its inherent bioactivity and physical-chemical adaptability [69], and due to its porous architecture, it promotes migration, proliferation and osteogenic and chondrogenic differentiation [13,70]. In studies reported by Comunian et al. and Ekizer et al. [43,64], who evaluated photobiomodulation in bone repair, it was verified that this modality enabled the improvement of neoformation and the quality of the formed bone tissue. This is because photobiomodulation promotes the increased synthesis of the number of osteocytes, collagen and DNA synthesis, proliferation and the differentiation of osteoblasts, in addition to cell metabolism [43,44]. Therefore, all these factors would also explain the better results obtained by the GXLEDG (GelMA + Ximenia + LED) group at 30 days.

## 4. Conclusions

The *Ximenia americana* L. stem extract incorporated into the GelMA showed satisfactory results, i.e., it accelerated bone repair in the first 15 days after the fracture. Regarding the association of biomaterial + LED, the group treated with GelMA + *Ximenia americana* L. + LED (GXLEDG) optimized the results by repairing and strengthening the injured bone region in 30 days. Histologically, it was shown that both therapies applied in the study (associated or not), caused greater filling by the bone trabeculae compared to the CG. In 30 days, the GXLEDG and GXG (GelMA + *Ximenia americana* L.) groups presented newly formed bone tissue that is clearly more compact with maturation to bone tissue with a cortical pattern. However, it is possible to observe that the *Ximenia americana* L. extract incorporated into the GelMA, together with the photobiomodulation from the LED, is a potentiator for bone repair in an animal model.

## Data Availability

The data presented in this study are available from the corresponding author upon request.
